# Early Oral Ovalbumin Exposure during Maternal Milk Feeding Prevents Spontaneous Allergic Sensitization in Allergy-Prone Rat Pups

**DOI:** 10.1155/2012/396232

**Published:** 2011-12-04

**Authors:** Adaweyah El-Merhibi, Kerry Lymn, Irene Kanter, Irmeli A. Penttila

**Affiliations:** ^1^Women's and Children's Health Research Institute, North Adelaide, SA, 5006, Australia; ^2^Discipline of Pediatrics, Department of Health Sciences, University of Adelaide, SA, 5005, Australia; ^3^Discipline of Medicine, Department of Health Sciences, University of Adelaide, SA, 5005, Australia

## Abstract

There are conflicting data to support the practice of delaying the introduction of allergenic foods into the infant diet to prevent allergy development. This study investigated immune response development after early oral egg antigen (Ovalbumin; OVA) exposure in a rat pup model. Brown Norway (BN) rat pups were randomly allocated into groups: dam reared (DR), DR pups challenged daily (days 4–13) with oral OVA (DR + OVA*c*), DR pups challenged intermittently (on day 4, 10, 12, and 13) with oral OVA (DR + OVA*i*), formula-fed pups (FF), and FF pups challenged daily with oral OVA (FF + OVA). Immune parameters assessed included OVA-specific serum IgE, IgG1, and IgA. Ileal and splenic messenger ribonucleic acid (mRNA) expression of transforming growth factor-beta (TGF-**β**1), mothers against decapentaplegic (Smad) 2/4/7, and forkhead box P3 (Foxp3) were determined. Ileum was stained for TGF-**β**1 and Smad4. *Results*. Feeding OVA daily to DR pups maintained systemic and local gut antibody and immunoregulatory marker mRNA responses. Systemic TGF-**β**1 was lower in DR + OVA*i* pups compared to DR and DR + OVA*c* pups. Feeding OVA to FF pups resulted in significantly greater OVA-specific IgE and IgG1, and lower IgA and TGF-**β**1 and Smad expression compared to DR pups. *Conclusions*. Early daily OVA exposure in the presence of maternal milk maintains immune markers associated with a regulated immune response, preventing early allergic sensitization.

## 1. Introduction

Allergic disease arises due to a complex interaction between genetic predisposition and environmental factors, breast or formula feeding and patterns of early microbial exposure [1–3]. The most common food allergies emerging in young infants are to egg and peanut antigens. Approximately 6–8% of children under three years of age are affected, with the incidence of these allergies increasing [4–7]. Food allergy to milk and eggs typically disappears by age three to five, however there are data to suggest that the natural history of food allergy may be changing and even food allergies, such as egg and milk, which we think of as typically transient are showing greater persistence into teenage and adult years [8, 9].

Antigen (allergen) stimulation of the mucosal immune system is thought to be critical for the development of oral tolerance. In early life, exposure to repeated doses of food antigens may help prime the developing immune response toward induction of oral tolerance [10]. The ability to develop tolerance to allergens also appears to coincide with the establishment of healthy gut colonization by commensal bacteria [11]. Failure to develop oral tolerance is thought to be associated with development of food-allergic disease. However, the mechanism(s) by which the normal intestinal immune system responds to food and its involvement in the development of food allergy remains unresolved. Understanding the mechanisms involved would allow for the potential to develop intervention strategies for the prevention of food allergy and also therapeutic treatments for infants who have already developed food allergy.

Oral tolerance to food antigens can be induced experimentally, but optimization of the dose used for sensitization is critical [12]. For example, induction of tolerance to peanut requires a significantly higher oral dose than for egg. Animals fed high doses of chicken OVA secrete more interleukin-4 (IL-4; associated with allergy) and less TGF-*β* (associated with tolerance) than those fed low doses, where more TGF-*β* and less IL-4 are produced [13]. There are only a few studies in neonates assessing timing of antigen exposure in inducing oral tolerance. In an animal model, Strobel et al. [14] have shown that oral OVA given in the first week of life to mice induces humoral as well as cell-mediated immunity [14]. In contrast, recent studies associate early antigen exposure with development of tolerance [15, 16]. More research is required to determine the optimum intervention strategy to promote oral tolerance.

Maternal milk cytokines, such as TGF-*β*2 and interleukin (IL-10) have the potential to regulate immune responses to food antigens and promote tolerance [17–23]. Although the relationship between breastfeeding and allergy prevention is controversial [24–26], there has recently been a growing interest in the role of breast milk in regulating immune response development to food antigens as new foods are introduced into the diet [16, 27].

During infancy, T helper 1 (Th1) immune response development is important in preventing persistent T helper 2 (Th2) responses and the subsequent promotion of allergic disease [3]. The maturation of naïve T cells into committed effector and regulator cells depends on complex interactions between antigen, immune cells, and the immediate cytokine environment. TGF-*β*, which predominantly signals through the Smad family of proteins, plays a major role in the development of T-cell lineage. TGF-*β* induces development of Foxp3^+^ T regulatory cells (Tregs) to promote tolerance [28, 29]. IL-4 together with TGF-*β* inhibits the generation of Foxp3^+^ Tregs by promoting Th cells that secrete IL-10, but which do not have regulatory function [30]. TGF-*β* in the local gut environment plays an important role in development of the infant immune response to food antigens as they are introduced into the diet [23, 31].

The interactions between breastfeeding and the timing of food antigen encounter are key factors which influence food allergy development [15, 32]. Currently there is a concern that delayed feeding until after 6 months (traditional weaning age) may program the developing immune response toward sensitization instead of tolerance [33, 34]. In countries where delayed feeding has been recommended, rates of food allergy have escalated, including a greater than 5-fold increase observed in food anaphylaxis in Australian children under 4 years of age [35]. The local intestinal environment plays an important role in regulating immune response development during introduction of food antigens. Since analysis of the local gut immune response during oral antigen introduction is not ethically feasible in infants, we assessed in an atopic rat pup model the developing immune response after daily early oral OVA exposure (continuous), as compared to intermittent (occasional) OVA exposure. In this *in vivo* study we focused on an early weaning time point (day 14). The developing immune response was assessed when OVA was introduced into the diet during a critical time in early life. Formula-fed groups were included as controls, as we have previously shown sensitization after early oral antigen feeding in formula-fed pups [16]. Egg ovalbumin was used as the target antigen to assess antigen-specific responses as it is one of the most common causes of food allergy in infants.

## 2. Materials and Methods

### 2.1. Animals

The BN rat has a naturally occurring genetic predisposition toward allergy development [36–39]. BN rats were bred and housed in the Animal Facility of the Child, Youth and Women's Health Services, Adelaide and experimentation was completed with approval from the Child, Youth and Women's Health Services Animal Ethics Committee.

### 2.2. Cannulation and Maintenance

The details of the artificial rat milk (formula) composition (Wombaroo Food Products, South Australia, Australia; Table 1 of Supplementary Material available doi 10.1155/2012/396232) and the procedure for artificial feeding have been previously described [16, 23]. We have also previously shown that the artificial rat milk (formula) does not contain active TGF-*β* [18, 40]. Briefly, at day 4 of age, rat pups in the formula fed groups were lightly anesthetized using forthane (Isoflurathane) and surgically implanted with a flexible i.g. cannula. Artificial rat milk was delivered to rat pups through a polyethylene line connected to the cannula using a multisyringe infusion pump (KDS220 multisyringe infusion pump; KD Scientific). We have demonstrated that changes in immune markers are directly attributed to the formula and not the surgical procedure [17].

### 2.3. Experimental Design

Rat dams were fed a standard non-purified diet which does not contain OVA (Ridley Agriproducts Pty Ltd, Victoria, Australia). Rat pups from 12 BN litters were randomly assigned to groups (*n* = 8/group). Each group (including the dam reared groups) were composed of a mix of pups taken from litters originating from a number of different dams. A daily or an intermittent oral OVA exposure regime was used. There was five feeding groups: dam reared pups (DR), DR pups receiving daily oral gavage (0.1 mL) of 10 mg OVA/day (OVA: Sigma-Aldrich, St.Louis, Mo, USA) from day 4–13 (DR + OVA*c*), DR pups receiving an initial oral gavage of OVA at day 4 followed by subsequent gavage with OVA on day 10, 12, and 13, (0.1 mL) of 10 mg OVA/day (DR + OVA*i*), formula-fed pups (FF), and FF pups receiving a daily oral gavage (0.1 mL) of 10 mg OVA/d (FF + OVA). Rat pups were killed at day 14 (prior to weaning).

Blood was collected by cardiac puncture and sera stored at −80°C. The spleen was removed, snap-frozen in liquid nitrogen, and stored at −80°C. The gastrointestinal tract was excised, and tissue from the ileum was isolated and either weighed and snap-frozen in liquid nitrogen for later RNA and protein analysis or fixed in 4% neutral buffered formaldehyde for 24 hour and transferred to 70% (v/v) ethanol for later processing.

### 2.4. IgE, IgG1, and IgA Analyses

Serum OVA-specific IgE and OVA-specific IgG1 were quantified by ELISA as previously described [23] but OVA was used for coating. Sera from the pups were diluted 5-fold for analysis in the ELISA assay. Standards and samples were added in duplicate and detected colorimetrically using 3,3′,5,5′ tetramethylbenzidine (TMB; Sigma-Aldrich Chemical Co., St. Louis, Mo, USA). The limits of detection for the OVA-specific IgE and OVA-specific IgG1 ELISA assays were 1.95 and 0.78 ng/mL, respectively. The plates were read with a Sunrise Magellan plate reader at 450 nm (Tecan Group Ltd, Mannedorf, Switzerland) and data expressed as ng immunoglobulin/mL sera.

IgA was quantified by ELISA using ileal tissue. Ileal protein lysates for use in the IgA ELISA were prepared as described in Tooley et al. [16]. Briefly, ileal protein lysates were prepared by adding a cocktail of protease inhibitors (Sigma-Aldrich Chemical Co., St. Louis, Mo, USA) to intestinal tissue (1 mL/100 mg tissue), which was then homogenized and centrifuged twice. Supernatants were collected, aliquoted, and stored at −80°C until analysed. Samples from pups for IgA analyses were diluted 1/2000 for DR groups and 1/5 for FF groups. The standard, purified rat IgA*κ*, capture antibody, mouse anti-rat IgA and the secondary, biotin mouse anti-rat IgA were all purchased from BD Biosciences (Franklin Lakes, NJ, USA). Briefly, 96-well plates (Greiner, Frickenhausen, Germany) were coated with 2 *μ*g/mL mouse anti-rat IgA in phosphate-buffered saline (PBS) overnight at 4°C. The wells were washed five times with wash buffer (PBS/0.05% Tween20) and then blocked for 1 hour at room temperature with 1% Polypep protein digest (Sigma-Aldrich Chemical Co., St. Louis, Mo, USA) in PBS. The samples and standards (purified rat IgA; standard range: 125 ng/mL to 1.95 ng/mL) were then added to the plate and incubated for 1 hour at room temperature. After incubation, the plates were washed five times and biotin mouse anti-Rat IgA was added (0.5 *μ*g/mL). Plates were incubated at room temperature for 1 hour and then washed six times. Following the final wash, a solution of ABC reagent (Vector Laboratories, Inc. Burlingame, Calif, USA) was added and the plates incubated for 30 minutes at room temperature. Plates were then washed six times; after washing TMB substrate was added to the wells for 30 minutes after which time the reaction was stopped using 50 *μ*L of 2 N HCl and read at an absorbance of 450 nm. The limit of detection for the IgA was 1.95 ng/mL. Data was expressed as ng immunoglobulin/g of tissue.

### 2.5. Real-Time PCR

RNA extraction from the spleen and ileum, cDNA synthesis, primer design, real-time PCR, and analysis were performed as previously described [16]. Primers for TGF-*β*1, Smad2, Smad4, *i*Smad7, and Foxp3 are provided in Table 1.

### 2.6. Histological Assessment

Immunohistochemical analyses of TGF-*β*1 and Smad4 were carried out on segments of the ileum. Four-micrometer sections were cut from paraffin-embedded tissue and placed on gelatin-coated slides. Sections were deparaffinized with xylene and rehydrated in graded ethanol in water. Sections were then placed in 10 mM citrate buffer (1.8 mM citric acid; 8.2 mM sodium citrate, pH 6.0) and subjected to heat-induced epitope recovery using microwave irradiation [41]. Sections were then cooled at room temperature for 30 minutes before staining.

For TGF-*β*1, sections were stained as described in Penttila et al. [40]. For Smad4 staining, tissue sections were first incubated with 5% normal horse serum/1% bovine serum albumin (5% NHS/1% BSA) in Tris buffered saline (TBS) for 30 minutes at room temperature to block nonspecific binding of the secondary antibody. The blocking antibody was then decanted and 100 *μ*L of anti-Smad4 IgG (8 *μ*g/mL; Santa Cruz Biotechnology Inc, Santa Cruz, CA, USA) was added, and the sections were incubated overnight at 4°C. After incubation the sections were then washed three times in TBS containing 0.05% Tween 20 (TTBS; 5 minutes/wash) and then incubated in 3% (v/v) hydrogen peroxide for 15 minutes at room temperature to quench endogenous peroxidase activity. The sections were then washed three times with TBS (5 minutes/wash) after which the secondary antibody (HRP conjugated donkey anti-mouse—3.2 *μ*g/mL; Jackson Immuno Research Laboratories, West Grove, PA. USA) was applied to the sections (100 *μ*L per section) and the sections incubated for 60 minutes at room temperature. The sections were then washed with TTBS (5 minutes/wash) two times followed by two washes with TBS (5 minutes/wash). For both TGF-*β*1 and Smad4 staining, immunohistochemistry reactions were visualized using a 3,3-diaminobenzidine (DAB) substrate plus enhancer (Invitrogen, Carlsbad, Calif, USA). After substrate development, sections were counterstained, dehydrated with graded ethanol, and mounted.

Control samples for TGF-*β*1 included sections incubated with normal chicken IgY (R&D Systems Inc, Minneapolis, MN, USA) or with antibody dilution buffer only. Control samples for Smad4 included sections incubated with 5% NHS/1% BSA (R&D Systems Inc, Minneapolis, MN, USA) or with the isotype control, mouse IgG1 (8 *μ*g/mL). Digital images of both TGF-*β*1 and Smad4 immunohistochemical sections (400x magnification) were taken and analysed using Image Pro Plus software, version 5.1 (Media Cybernetics, Bethesda, Md, USA).

### 2.7. Statistical Analyses

All data were expressed as the mean + standard error of the mean (SEM). Data was assessed for Normality before analysis. OVA-specific IgE and IgG1 and TGF-*β*1, Foxp3, Smad2, Smad4, and *i*Smad7 mRNA expression data were evaluated utilizing a nonparametric one-way ANOVA (Kruskal-Wallis) followed by a Dunn's Multiple Comparisons post hoc test. Differences were considered significant at *P* < 0.05. All statistical analyses and comparisons were made using GraphPad Prism software, version 3 (GraphPad Software Inc, San Diego, Calif, USA).

## 3. Results

### 3.1. Bodyweight Change

Feeding OVA to either DR or FF pups did not affect body weight gain at day 14 (data not shown).

### 3.2. OVA-Specific IgE, OVA-Specific IgG1 and IgA

**Figure 1:**
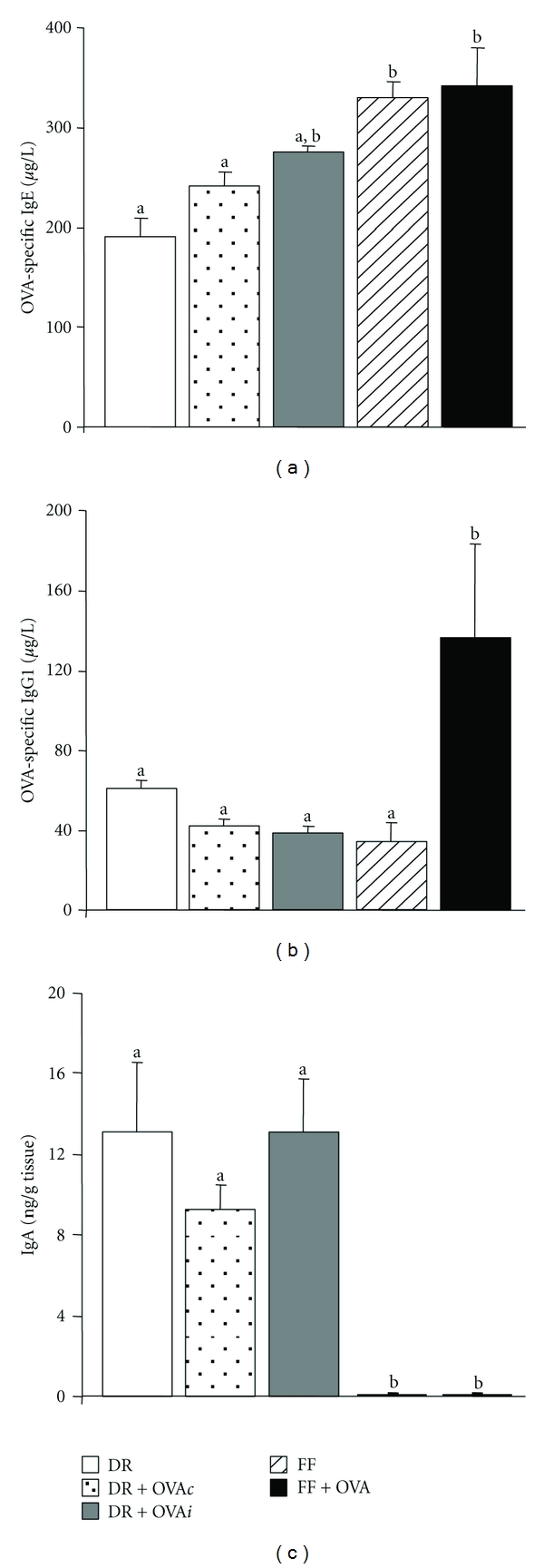
OVA-specific IgE, OVA-specific IgG1, and ileal IgA after oral OVA commenced at day 4 in DR or FF rat pups. Bars are mean + SEM, *n* = 8/group. Means without a common letter differ, *P* < 0.05.

OVA given during formula feeding resulted in a significantly increased OVA-specific IgE titer compared with the DR and DR + OVA*c* groups (*P* < 0.05; Figure 1(a)). Serum OVA-specific IgG1 was also significantly increased in the FF + OVA group (*P* < 0.05) compared with the DR, DR + OVA*c*, DR + OVA*i*, and FF groups (Figure 1(b)). Importantly, OVA-specific IgG1 titers did not differ significantly between the DR groups regardless of oral OVA exposure. IgA was significantly greater in the DR, DR + OVA*c*, DR + OVA*i* groups (*P* < 0.01) and barely detectable in the FF and FF + OVA groups (Figure 1(c)). IgA levels did not differ between the DR groups.

### 3.3. TGF-*β*1 and Smad mRNA Expression in Spleen and Ileum

**Figure 2:**
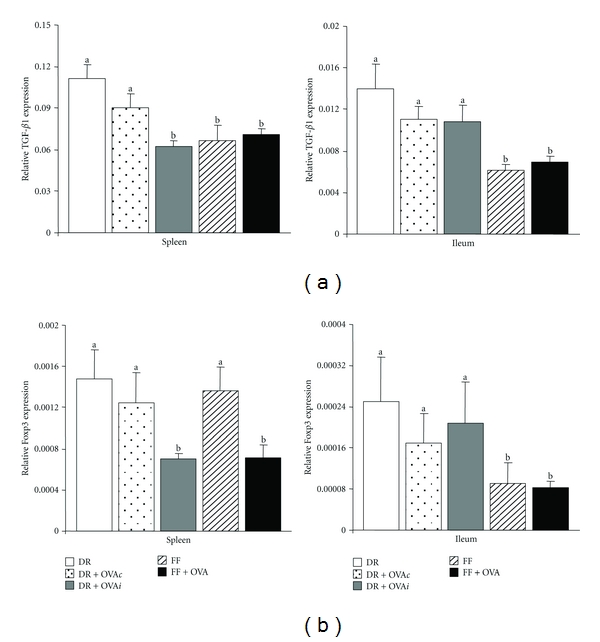
Splenic and ileal cytokine mRNA expression in DR and FF pups at day 14 with or without daily/intermittent treatment with OVA. TGF-*β*1 mRNA (a) and Foxp3 mRNA (b) as determined by real-time PCR. Bars are mean + SEM, *n* = 7-8. Means without a common letter differ, *P* < 0.05.

TGF-*β*1 mRNA expression in the spleen was significantly greater in the DR and DR + OVA*c* groups compared with the DR + OVA*i*, FF and FF+OVA groups (*P* < 0.05; Figure 2(a)). Splenic TGF-*β*1 mRNA expression did not differ significantly between the DR and DR + OVA*c* groups. TGF-*β*1 mRNA expression in the ileum was significantly greater in all DR groups regardless of OVA exposure compared with the FF and FF + OVA groups (*P* < 0.05; Figure 2(a)); however there were no significant differences in ileal TGF-*β*1 mRNA expression between the DR groups. Foxp3 mRNA expression in the spleen was significantly greater in the DR, DR + OVA*c*, and FF groups compared with the DR + OVA*i* and FF + OVA groups (*P* < 0.05; Figure 2(b)). Expression did not differ significantly between the DR, DR + OVA*c*, and FF groups. Although the mRNA expression of Foxp3 in the ileum did not differ between the DR groups, expression was significantly greater in DR groups compared with the FF and FF + OVA groups (*P* < 0.05; Figure 2(b)).

**Figure 3:**
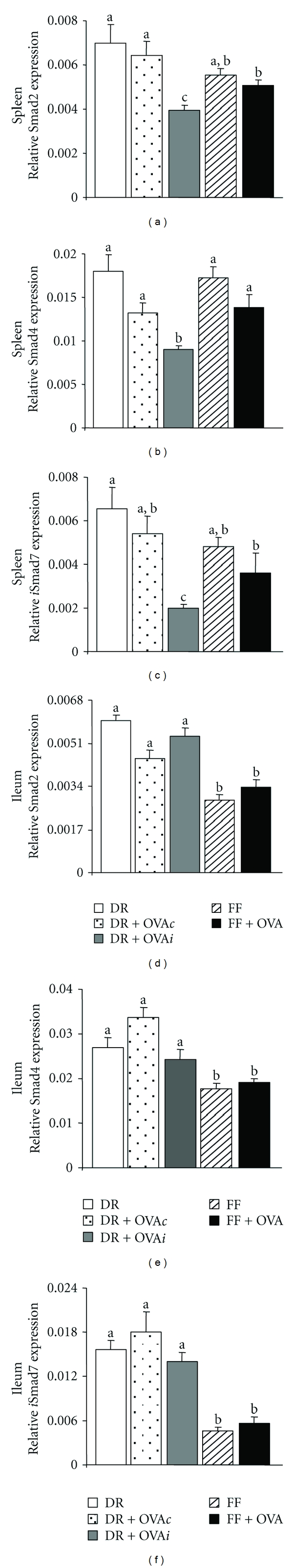
Splenic and ileal mRNA expression of Smad pathway genes in DR and FF pups at day 14 with or without daily/intermittent treatment with OVA. Smad2, 4, and 7 mRNA levels in spleen (a, b, and c) and Smad2, 4 and 7 mRNA levels in ileum (d, e, and f) as determined by real-time PCR. Bars are mean + SEM, *n* = 7-8. Means without a common letter differ, *P* < 0.05.

The Smad pathway was also investigated by analyzing the mRNA expression of Smad2, Smad4, and *i*Smad7 in the spleen and ileum. In the spleen, Smad2 mRNA expression was significantly greater in DR group compared with the DR + OVA*i* and FF + OVA groups (*P* < 0.05; Figure 3(a)). Expression of splenic Smad2 mRNA did not differ significantly between the DR, DR + OVA*c*, and FF groups. Smad 4 mRNA expression in the spleen was significantly greater in DR and FF groups compared with the DR + OVA*i* group (*P* < 0.05; Figure 3(b)). No significant difference in Smad4 mRNA expression was observed between the DR, DR + OVA*c*, FF, and FF + OVA groups. *i*Smad7 mRNA expression in the spleen was significantly greater in the DR and DR + OVA*c* groups compared with the DR + OVA*i* and FF + OVA groups (*P* < 0.05; Figure 3(c)). No significant difference in* i*Smad7 mRNA expression in the spleen was observed between DR, DR + OVA*c*, and FF rats. In the ileum, Smad2, Smad4 and *i*Smad7 mRNA expression was significantly greater in the DR groups regardless of OVA exposure compared with FF and FF + OVA groups (*P* < 0.05; Figures 3(d), 3(e), and 3(f)). There were no significant differences in Smad2, Smad4, and *i*Smad7 mRNA expression in the ileum between the DR, DR + OVA*c*, and DR + OVA*i* groups.

### 3.4. TGF-*β*1 and Smad4 Protein Expression in the Ileum

**Figure 4:**
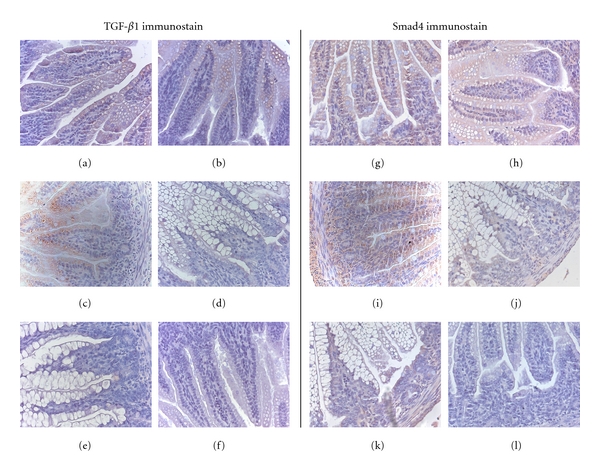
Mucosal immunolocalization of TGF-*β*1 and Smad4 in DR and FF pups at day 14 with or without daily/intermittent treatment with OVA. Representative images are shown for all groups. Positive staining is indicated as a brown color. TGF-*β*1 (a–f: DR (a), DR + OVA*c* (b), DR + OVA*i* (c), FF (d), FF + OVA (e), and negative control (f)) and Smad4 (G-L: DR (g), DR + OVA*c* (h), DR + OVA*i* (i), FF (j), FF + OVA (k), and negative control (l)).

TGF-*β*1 staining was mainly localized to the enterocytes and occasional individual cells in the villus lamina propria of the ileum (Figures 4 (a), 4(b), 4(c), 4(d), and 4(e)). No TGF-*β*1 staining was evident at the base of the villus in the crypts or the surrounding lamina propria. Staining of Smad4 was localized throughout the enterocytes of the villi and in cells of the lamina propria in all rat groups (Figures 4(g), 4(h), 4(i), 4(j), and 4(k)). Smad4 was not detected in goblet cells or the longitudinal layer of smooth muscle. TGF-*β*1 and Smad4 staining was consistently more abundant in the DR groups regardless of OVA exposure when compared to staining in sections from FF or FF + OVA rats. No background staining was detected in negative controls (Figures 4(f) and 4(i)).

## 4. Discussion

We investigated the immune response profile after early oral OVA exposure in DR and FF rat pups. Early oral OVA exposure in rat pups, regardless of the dosage regime, in the presence of maternal milk maintained a similar immune response profile to that observed in DR unchallenged rats, with low levels of circulating OVA-specific IgE; and IgG1. In contrast to the low OVA IgG1 response seen in the rat pups fed formula alone (no OVA challenge), the IgE response to OVA was high (not significantly different from that seen in the OVA challenged formula fed rat pups). We have previously shown that formula feeding induces an overall increase in total serum IgE, this increased IgE response may contain cross-reactive antibodies to OVA [16]. Formula fed groups were only included as controls in this study, as we have previously shown sensitization after early oral antigen feeding in formula-fed pups [16]. The results seen for OVA-specific IgG1 are similar to our previous published data relating to feeding cow's milk allergen, *β*-lactoglobulin (BLG), where we showed that sensitization was prevented in maternal-milk-fed pups given oral BLG early in life. Importantly we showed that this regulated immune profile persisted into postweaning age [16, 23]. In contrast, immune activation and allergy development resulted when BLG was fed in the presence of formula [16].

TGF-*β*s are an important family of growth factors involved in maintaining homeostasis in the intestine, regulating inflammation and allergy development and promoting oral tolerance development in infants [31]. TGF-*β* is the predominant cytokine present in human and rodent milk [40, 42]. TGF-*β* predominately signals through the Smad protein family. Smad2 and Smad3 are phosphorylated after activation of TGF-*β* receptors, forming a complex with Smad4. Once translocated into the nucleus, this complex then binds to the Smad binding element in the promoter region of TGF-*β* target genes and regulates transcriptional responses in conjunction with DNA-binding partners [43, 44]. By also assessing Smad genes involved in the pathway we have been able to further elucidate the function of TGF-*β*1 in the development of immune responses in the gut when OVA was introduced. In the DR + OVA*c* group, TGF-*β*1 mRNA expression in both the spleen and the local gut environment did not differ significantly from that in unchallenged DR rats. However, DR rats receiving oral OVA intermittently displayed decreased TGF-*β*1 and Smad2, Smad4, and *i*Smad7 mRNA expression in the spleen. Collectively, we have shown that the DR + OVA*i*, FF, and FF + OVA groups exhibited the greatest systemic impairment of the TGF-*β*1/Smad pathway with lower mRNA expression of the TGF-*β*1/Smad genes. However, in the intestine, which is the first site of exposure to food antigens we observe a different pattern of expression. DR rats exposed to OVA intermittently maintained a TGF-*β*1/Smad mRNA expression profile similar to the unchallenged DR group. One possible explanation is that external sources of TGF-*β*, provided by maternal milk, are sufficient to maintain the expression of these genes in the local gut environment. Our current data also shows that in the ileum of FF rats, with or without OVA exposure, lower levels of all Smad mRNA's were present when compared to the DR groups. High levels of TGF-*β* are present in rat milk during early lactation, with the highest levels detected just after birth. TGF-*β* levels then decrease toward weaning. In contrast in maternal-fed rat pups, the number of TGF-*β*1-producing cells and mRNA in the intestine is low after birth, but levels increase over the weaning period [40]. As TGF-*β* is essential for maintaining homeostasis in the intestine and promotion of T regulatory cells, our data suggests that the local gut environment in FF pups is impaired with regard to the potential for developing regulated immune responses to food antigens. We have shown in previous studies that when BN rat pups are fed a formula supplemented with physiological levels of TGF-*β*, markers associated with allergy development are reduced and the immune response profile to the cow's milk allergen, BLG, is not significantly different to that seen in unchallenged maternal-milk-fed pups. This regulated immune response profile extended out to postweaning ages, highlighting the importance of TGF-*β* in developing and preventing sensitization to food antigens [23].

TGF-*β*1 signals are controlled by inhibitory *i*Smads, predominantly *i*Smad7 [43, 44]. We have shown, particularly in the ileum, that TGF-*β*1 and *i*Smad7 mRNA levels maintain a homeostatic balance, possibly by forming a negative feedback loop. It has been documented that the transcription of *i*Smad7 can be turned on by TGF-*β* itself in the TGF-*β*/Smad signalling pathway [45]. This suggests that even in the presence of early oral antigen challenge the mucosal immune system can develop and maintain such regulatory mechanisms.

TGF-*β* signaling promotes T-cell tolerance and helps maintain normal homeostasis throughout the lifespan. TGF-*β* preferentially increases IgA antibody responses by directing isotype switching to IgA in Peyer's patches [46]. Ogawa et al. [47] showed in a study of newborn infants during their first month of life that an increase of serum IgA correlated with levels of both TGF-*β*1 and TGF-*β*2 in maternal colostrum [47]. Our data supports the role of TGF-*β* in regulating IgA levels during early food introduction in the presence of maternal milk. We have shown that early oral OVA exposure in the presence of maternal milk, as compared to formula, maintained IgA levels. In the FF groups, IgA levels were only slightly above the detection limit of the assay.

As well as being involved in epithelial growth, IgA production, DC maturation, and Treg cell differentiation, TGF-*β*s inhibit inflammation and regulate inflammatory responses in the intestine [17, 48–51]. In the adult intestine, TGF-*β*1 is the predominant isotype present in epithelial and lamina propria cells [52]. We assessed the localization pattern of both TGF-*β*1 and Smad4 in the intestine of DR and FF rats with or without antigen exposure. More abundant staining of TGF-*β*1 and Smad4 was observed in all the DR groups, regardless of antigen exposure compared with the FF groups. The histology supports our TGF-*β*1 and Smad4 mRNA expression data in the ileum and again highlights the potential for sensitization in FF rat pups. We have demonstrated in the local gut environment that formula feeding early in life results in an overall suppression of TGF-*β*1 and the signaling genes involved in its pathway, namely, Smad2, Smad4, and *i*Smad7.

TGF-*β* is also required for induction of Tregs, which play a critical role in maintaining immune homeostasis in the intestine. Foxp3^+^ (CD4^+^CD25^+^Foxp3^+^) regulatory cells are necessary for the development of oral tolerance [53–56]. Foxp3 mRNA expression was maintained in the ileum of DR rats receiving OVA daily with expression levels similar to that seen in the ileum of unchallenged DR rats. Ileal Foxp3 mRNA expression in the DR + OVA*i* group did not differ from the DR or DR + OVA*c* groups but a decrease in splenic Foxp3 mRNA was observed in the DR + OVA*i* group. A decrease in Foxp3 mRNA expression was also observed in both the ileum and spleen of FF rat pups receiving OVA. We have previously shown that this decrease in FoxP3 mRNA expression was also noted in the mesenteric lymph node of FF BN rat pups at day 14, and that the frequency of Foxp3^+^ cells was greater in maternal-fed BN rat pups receiving a continuous dose of BLG [16]. The role of Foxp3^+^/CD25^+^/CD4^+^ Tregs in development of food allergy is at present unclear. It has been shown that Foxp3^+^cells are present in the intestine of food allergic children, but Foxp3 transcription levels are low [57]. In contrast, other studies report lower expression of Foxp3 and defects in transcription [55]. It has been shown that repeated small doses of antigens are necessary for the development of oral tolerance mediated by Treg cells [58]. Our results suggest that continuous as opposed to intermittent antigen exposure in the presence of maternal milk maybe required to promote Treg cells and Foxp3 expression in the periphery. A daily repeated exposure to OVA as compared to an intermittent (occasional) exposure may allow the immature immune system time to “practice” and therefore help to prime for development of a regulated immune response to prevent sensitization, potentially enhancing later tolerance development.

In infants with a predisposition toward allergy development, delaying the feeding of solids until after 6 months may program the developing immune response toward sensitization [15, 59]. Factors such as the duration of exclusive breastfeeding, timing of introduction, and the type of other foods (allergens) in the diet are also thought to influence the switch between tolerance and sensitization [15]. In support of this, in a previous time course study of early cow's milk allergen exposure (BLG was commenced at day 4 of life) we showed a reduction in the levels of markers associated with allergy development at day 10, 14, and 21 of life after BLG exposure in the presence of maternal milk [16]. In our current study assessing an early weaning time point (day 14 of life) we show an upregulation of the levels of markers associated with immuno-regulatory mechanisms after early OVA exposure. Daily but not intermittent oral OVA exposure commenced on day 4 during maternal milk feeding created an immune environment with the potential to decrease sensitization to food antigens. Foxp3 mRNA expression, TGF-*β*1 mRNA and protein expression, and expression of the Smad genes involved in TGF-*β* signaling were maintained in both the microenvironment of the gut and the periphery. Early regular exposure to food antigens (OVA) in the presence of maternal milk in early life maintains immune regulatory mechanisms preventing allergic sensitization.

## Supplementary Material

Supplementary Table 1 outlines the composition of the rat milk replacer which was developed to closely represent maternal rat milk in composition (Wombaroo Food Products SA, Australia). The formula provides artificially reared pups with nutrients (energy, lipids and protein) for growth.

## Figures and Tables

**Table 1:** 

Gene	Forward primer	Reverse primer
TGF-*β*1	TGCGCCTGCAGAGATTCAAGTCAA	AAAGACAGCCACTCAGGCGTATCA
Smad2	TGAGCTTGAGAAAGCCATCA	TGTGTCCCACTGATCTACCG
Smad4	GGCATTGGTGTAGACGACCT	GGGGTTTCTTTGATGCTCTG
*i*Smad7	GCAGCAGTTACCCCATCTTC	TGATGGAGAAACCAGGGAAC
FoxP3	CCACACCTCCTCTTCTTCCTT	TGACTAGGGGCACTGTAGGC
Cyclophilin A	GGTTGGATGGCAAGCATGTG	TGCTGGTCTTGCCATTCCTG
