# Supplementary material for: Early Oral Ovalbumin Exposure during Maternal Milk Feeding Prevents Spontaneous Allergic Sensitization in Allergy-Prone Rat Pups
Source: Clin Dev Immunol. 2011 Dec 4;2012:396232. doi: 10.1155/2012/396232 (PMC3235444; doi:10.1155/2012/396232)
Supplement: Supplementary file 1 — Supplementary Table 1 outlines the composition of the rat milk replacer which was developed to closely represent maternal rat milk in composition (Wombaroo Food Products SA, Australia). The formula provides artificially reared pups with nutrients (energy, lipids and protein) for growth. [file 396232.f1.pdf]

Supplemental Table 1: Approximate composition of the Rat Milk Replacer

Rat Milk Replacer	
**Metabolizable Energy**	6500kJ
g/L dry matter	
**Solids**	260
**Lipid**	119
**Protein**	87
**Lactose**	33
**Vitamins & Minerals**	46.3
**β-Lactoglobulin**	2.45
**Lipids**	
**C16:0**	20
**C18:0**	9
**C18:1(n-9)**	54
**C18:2(n-6)**	13
**C18:3(n-3)**	6
**C20:4(n-6)**	0.040
**C20:5(n-3)**	0.068
**C22:6(n-3)**	0.040
